# Mutation in the C-Di-AMP Cyclase *dacA* Affects Fitness and Resistance of Methicillin Resistant *Staphylococcus aureus*

**DOI:** 10.1371/journal.pone.0073512

**Published:** 2013-08-27

**Authors:** Vanina Dengler, Nadine McCallum, Patrick Kiefer, Philipp Christen, Andrea Patrignani, Julia A. Vorholt, Brigitte Berger-Bächi, Maria M. Senn

**Affiliations:** 1 Institute of Medical Microbiology, University of Zurich, Zurich, Switzerland; 2 Sydney Emerging Infectious Diseases and Biosecurity Institute (SEIB), University of Sydney, Sydney, Australia; 3 Institute of Microbiology, ETH Zurich, Zurich, Switzerland; 4 Functional Genomics Center Zurich, University/ETH Zurich, Zurich, Switzerland; Rockefeller University, United States of America

## Abstract

Faster growing and more virulent strains of methicillin resistant *Staphylococcus aureus* (MRSA) are increasingly displacing highly resistant MRSA. Elevated fitness in these MRSA is often accompanied by decreased and heterogeneous levels of methicillin resistance; however, the mechanisms for this phenomenon are not yet fully understood. Whole genome sequencing was used to investigate the genetic basis of this apparent correlation, in an isogenic MRSA strain pair that differed in methicillin resistance levels and fitness, with respect to growth rate. Sequencing revealed only one single nucleotide polymorphism (SNP) in the diadenylate cyclase gene *dacA* in the faster growing but less resistant strain. Diadenylate cyclases were recently discovered to synthesize the new second messenger cyclic diadenosine monophosphate (c-di-AMP). Introduction of this mutation into the highly resistant but slower growing strain reduced resistance and increased its growth rate, suggesting a direct connection between the *dacA* mutation and the phenotypic differences of these strains. Quantification of cellular c-di-AMP revealed that the *dacA* mutation decreased c-di-AMP levels resulting in reduced autolysis, increased salt tolerance and a reduction in the basal expression of the cell wall stress stimulon. These results indicate that c-di-AMP affects cell envelope-related signalling in *S. aureus*. The influence of c-di-AMP on growth rate and methicillin resistance in MRSA indicate that altering c-di-AMP levels could be a mechanism by which MRSA strains can increase their fitness levels by reducing their methicillin resistance levels.

## Introduction

Infections with methicillin resistant *Staphylococcus aureus* (MRSA) have severely impaired treatment outcomes and are cost intensive for the healthcare system. MRSA contain a staphylococcal cassette chromosome *mec* (SCC*mec)* element harbouring the *mecA* gene that codes for an alternative penicillin binding protein, PBP2a, which confers resistance to beta-lactams, the antibiotic class of first choice for treating *S. aureus* infections. Initially, MRSA were restricted to healthcare settings with high antibiotic pressure. These healthcare-associated (HA)-MRSA strains generally displayed low heterogeneous resistance profiles, whereby only small subpopulations could survive at high beta-lactam concentrations. Over time, HA-MRSA strains possessing very high beta-lactam minimum inhibitory concentrations (MICs) emerged, such as E-MRSA 16 (ST32-MRSA-II) or the Iberian clone (ST247-MRSA-I), which successfully spread in hospitals all over the world (for reviews see [1], [2]). Over the last fifteen years MRSA have started to spread in the community [3]. These so-called community-acquired (CA)-MRSA are characteristically fitter and more virulent than HA-MRSA strains (for reviews see [4], [5]). CA-MRSA strains, such as USA300, are capable of infecting healthy individuals without obvious risk factors [3]. CA-MRSA typically have relatively low oxacillin MICs, but their heterogeneous resistance profiles include higher-resistant subpopulations of bacteria that can cause treatment failure. CA-MRSA-like clones are increasingly displacing conventional, previously successful, HA-MRSA clones in hospitals [6].

Methicillin resistance does not only depend on PBP2a production but it is also affected by the genetic background of a strain [7] and factors influencing methicillin resistance, previously called *fem* (factors essential for methicillin resistance) or *aux* (auxiliary) factors [8], [9], [10]. These factors are often directly or indirectly involved in cell envelope biosynthesis and turnover. Examples of *fem/aux* factors linked to the cell envelope include cell wall biosynthesis enzymes like GlmM [11], MurE [12], MurF [13], FemABX [14], PBP2 [15], PBP4 [16]; and wall teichoic acid biosynthesis enzymes including TagO/TarO [17], the *dlt* operon [18] and the wall teichoic acid ligase MsrR [19], [20]. *Fem/aux* factors indirectly connected or with no obvious connection to the cell envelope include regulators like SigB [21], SpoVG [22], *agr* [23], SarA [23], XdrA [24], CcpA [25], SecDF [26] or two component systems like VraSR [27], [28].

Recently, a new second messenger, cyclic diadenosine monophosphate (c-di-AMP) was shown to influence methicillin resistance in *S. aureus* [29]. Second messengers are small molecules such as the nucleotides cAMP, cGMP, c-di-GMP or (p)ppGpp that regulate various cellular processes including virulence, biofilm formation, motility and the cell cycle. Levels of the nucleotide (p)ppGpp, involved in the stringent response, were also recently discovered to influence beta-lactam resistance [30]. Regulation by nucleotides can occur at transcriptional, translational and post translational levels (for reviews see [31], [32]). c-di-AMP is synthesized by condensation of two ATP molecules by diadenylate cyclase domain (DAC) proteins and was first identified by Witte *et al*. in *Bacillus subtilis* in 2008 [33]. In contrast to *B. subtilis*, which has three different proteins with DAC domains, *S. aureus* possesses only one DAC domain protein, DacA. DacA is claimed to be essential and shows a similar structure to the *B. subtilis* DAC domain protein CdaA (previously called YbbP) containing a transmembrane domain and the DAC domain [29]. Additionally, the genetic regions of *S. aureus* and *B. subtilis* have a similar organisation forming the three-gene operons *dacA-ybbR-glmM* and *cdaA-cdaR-glmM*, respectively, including the phosphoglucosamine mutase *glmM and* the recently identified c-di-AMP regulator *cdaR* or its *S. aureus* homologue *ybbR* [11], [34]. However, in *B. subtilis* the glucosamine-6-phosphate synthase gene *glmS* is located directly downstream of *glmM* and forms an additional large transcript *cdaA-ybbR-glmM-glmS* [34]. In contrast, *glmS* in *S. aureus* is separated from *glmM* by about 14 kb, which contains the cell wall associated protein *fmtB* (also called *mrp*) and mannitol synthesis and export genes [35]. A larger transcript including *fmtB* but not *glmS* has been detected in *S. aureus (dacA-ybbR-glmM-fmtB)* [36]. In *B. subtilis* the protein encoded directly upstream of the diadenylate cyclase, CdaR (previously called YbbR), is a c-di-AMP synthase regulator which was found to specifically stimulate c-di-AMP production by CdaA [34]. Mehne and colleagues suggested that the same positive regulation could occur by YbbR of *S. aureus* since DacA and CdaA share several common features, as described above. The cellular level of c-di-AMP is further influenced by degradation of c-di-AMP to 5’-pApA by the phosphodiesterase GdpP [29], [37]. In several studies, mutations in *gdpP* were found to increase resistance or tolerance to beta-lactam antibiotics in *B. subtilis, Listeria monocytogenes* and *S. aureus* by as yet unknown mechanisms [29], [38], [39], [40], [41], [42]. Besides antibiotic resistance, c-di-AMP affects cell envelope homeostasis in both *B. subtilis* and *S. aureus* [29], [40]. In *S. aureus* a 15-fold increased c-di-AMP level led to increased peptidoglycan cross-linking and could compensate for the absence of lipoteichoic acids [29]. Interestingly, c-di-AMP secreted from *L. monocytogenes* triggers a host type I interferon response; a c-di-AMP dependent type I interferon response was also detected in the Gram-negative *Chlamydia trachomatis* [43], [44]. Furthermore, a *gdpP* mutation in *Lactococcus lactis* led to salt hypersensitivity and heat resistance [45].

Very recently the first c-di-AMP receptors were identified [46], [47]. One is the TetR-like transcription factor DarR of *Mycobacterium smegmatis* [47] and four receptor proteins were identified in *S. aureus*; the potassium transporter-gating component KtrA, a predicted cation/proton antiporter CpaA, a PII-like signal transduction protein PstA and a histidine kinase KdpD [46]. However, it remains unclear how these c-di-AMP receptors influence cell envelope homeostasis and beta-lactam resistance, indicating that there might be more receptors.

Even though there has been extensive research on methicillin resistance, the mechanisms governing heterogeneous and homogeneous resistance in MRSA are not completely understood yet. In our study we analysed an isogenic MRSA strain pair differing in fitness and methicillin resistance levels. Whole genome sequencing identified only one single nucleotide polymorphism (SNP) which was in the diadenylate cyclase gene *dacA*. Markerless allelic replacement was used to confirm that this mutation was responsible for the transition from homogeneous to heterogeneous resistance and the associated faster growth rate. Quantification of cellular c-di-AMP levels indicated that phenotypic changes were caused by decreased c-di-AMP, due to the *dacA*-SNP.

## Materials and Methods

### Bacterial strains and growth conditions

The strains and plasmids used in this study are listed in Table 1. Bacteria were grown at 37°C in Luria Bertani (LB) broth (Difco Laboratories, Detroit, MI, USA), shaking at 180 rpm with a 1:5 culture to air ratio, or on LB agar plates unless indicated differently. Optical density (OD) was measured at 600 nm. Media were supplemented with the following antibiotics when appropriate: 10 µg/ml tetracycline (Sigma, St. Lousis, MO, USA), 10 µg/ml chloramphenicol (Sigma), 100 µg/ml ampicillin (Sigma), 200 ng/ml anhydrotetracycline (Sigma) or various concentrations of oxacillin (InfectoPharm, Heppenheim, Germany).

**Table 1:** Strains and plasmids.

Strain or plasmid name	Relevant genotype and/or phenotype	Source or reference
**Strains**		
***S. aureus***		
RN4220	Restriction-deficient derivative of NCTC 8325-4	[68]
BB255	NCTC8325 derivative, FK268 cured from plasmid pI524 by ethidium bromide treatment, ST8, Mc^S^	[53]
RA120	BB255 containing SCC*mec* type I, Mc^R^	[54]
ME51	RA120 derivative with increased fitness isolated from competition experiments, mutation in DacA (Gly206Ser), Mc^R^	[54]
COL	Clinical HA-MRSA, ST250, Mc^R^	[9]
BB255::*dacA-SNP*	BB255 with mutation in DacA (Gly206Ser), Mc^S^	This study
RA120::*dacA*-SNP	RA120 with mutation in DacA (Gly206Ser), Mc^R^	This study
COL::*dacA-SNP*	COL with mutation in DacA (Gly206Ser), Mc^R^	This study
***E. coli***		
DH5α	F^-^ φ80d/acZ*Δ*M15 *recA1*	Invitrogen
**Plasmids**		
pKOR1	*S. aureus-E. coli* shuttle vector, *ori* pAMα1, *ori* ColE1, *E. coli* Am^r^, *S. aureus* Cm^r^	[48]
pKOR1-*dacA*-SNP	pKOR1 construct containing the *dacA* gene with 1000-bp upstream and 1020-bp downstream regions carrying a mutation leading to Gly206Ser substitution in DacA	This study
p*sas016*p*-luc*+	pBUS1 containing the *sas016* promoter-luciferase reporter gene fusion	[67]

Abbreviations: Am, ampicillin; Cm, chloramphenicol; Mc, methicillin; Tc, tetracycline; r, resistant; s, susceptible; ST, sequence type.

### Whole genome sequencing and data analysis

Genome sequencing of strains RA120 and ME51 was performed by GATC Biotech AG (Konstanz, Germany) on an Illumina HiSeq 2000 (Illumina Inc., Saffron Walden, United Kingdom). Mapping of the 31-bp reads of a 200-bp paired-end library against the reference genome NCTC8325 and SNP calling was performed with the CLC Genomics workbench 4.8 (CLC Bio, Aarhus, Denmark), resulting in 63-fold mean depth coverage for RA120 (6’135’387 reads) and 83-fold mean depth coverage for ME51 (7’658’818 reads). The parent strain BB255 was sequenced using a PacBio RS SMRT sequencer (Pacific Biosciences Inc., Menlo Park, CA, USA) at the Functional Genomics Center Zurich. DNA from standard phenol/chloroform/isoamyl alcohol extractions was purified over DNeasy Mini Spin Columns (Qiagen, Düsseldorf, Germany). Two 250-bp libraries and one 6-kb library were prepared with the PacBio C1 chemistry kits provided by Pacific Biosciences according to the manufacturers’ recommendations. The library was quality inspected and quantified on the Agilent Bioanalyzer (Agilent Technologies, Santa Clara, CA, USA) and on a Qubit Fluorometer (Life technologies Europe, Zug, Switzerland), respectively. After the run, sequencing reports were generated via the SMRT portal to assess the adapter dimer contamination, the sample loading efficiency, the obtained average read length and the number of filtered sub-reads. Mapping of the sub-reads against the reference genome NCTC8325 and SNP calling was performed automatically via the SMRT portal and confirmed using CLC Genomics Workbench 4.8. A 174-fold mean depth coverage from 1’978’548 reads with an average read length of 199 bp from the 250-bp libraries and 126’945 reads with an average read length of 981 bp including reads reaching up to 17’000 bp from the 6-kb library were achieved. The sequencing data was submitted to the European Nucleotide Archive (ENA) under the study accession number PRJEB400 and the sample accession numbers ERS251419 (BB255), ERS251420 (RA120) and ERS251421 (ME51).

### Resequencing and construction of mutant strains

The identified SNP in *dacA* was confirmed by Sanger sequencing with the primers 2407.F (TTATTTCACACCTTTCTTTTGAAAG) and 2407.R (ATGGATTTTTCCAACTTTTTTCAAA) at Mircosynth (Balgach, Switzerland). The pKOR1 system developed by Bae & Schneewind was used to insert the mutation into *dacA* by homologous recombination [48]. The *dacA* gene, with additional 1000-bp upstream and 1020-bp downstream regions, was amplified from genomic DNA of strain ME51 using primers attB1-2407-upF (GGGG ACAAGTTTGTACAAAAAAGCAGGCT CGACACCTCTTACTCCGTCT) and attB2-2407-downR (GGGG ACCACTTTGTACAAGAAAGCTGGGT ACCAACAGCAATTAGATATG) and the fragment was recombined into the pKOR1 plasmid. Markerless allelic replacement was then performed as previously described [48].

### Oxacillin population analysis profiles and determination of oxacillin minimum inhibitory concentration (MIC)

Antibiotic resistance profiles were determined by plating appropriate dilutions of an overnight culture, ranging from undiluted to 10^−7^, on increasing concentrations of oxacillin. Plates were incubated at 37°C and colony forming units per ml (CFU/ml) were determined after 48 hours. Experiments were performed at least three times and representative data are shown. MICs were determined using Etest® strips (BioMérieux, Marcy l’Etoile, France) on Mueller-Hinton plates swabbed with an inoculum of 0.5 McFarland and incubated at 37°C for 24 h. MICs tests were performed at least three times.

### Autolysis

Spontaneous and induced autolysis were determined as previously described [26]. Briefly, cells were grown to OD 0.7, washed with 0.85% NaCl and resuspended in 0.01 M Na-phosphate buffer pH 7. The OD was adjusted to 0.7, cultures were incubated at 37°C and decreases in OD were measured over time. For induced autolysis 0.01% Triton X-100 (Fluka, Buchs, Switzerland) was added to the phosphate buffer after washing. Values shown represent the means and standard deviation from three independent experiments. For statistical analysis of individual time points Student’s *t*-tests were performed and for comparison of whole data sets two-way Anova was applied.

### PBP2a and PBP4 Western blot

*S. aureus* membrane fractions were obtained as described by Quiblier *et al*. [26] and PBP2a Western blot analyses were performed as described by Ender at al. [49]. Briefly, cells were sampled by centrifugation and pellets were frozen in liquid nitrogen then resuspended and lysed with lysostaphin, lysozyme and DNase in SSM buffer (500 mM sucrose, 2 mM malate, 20 mM MgCl_2_, pH 6.8). After five cycles of freezing and thawing the membrane fraction was obtained by centrifugation at 20’000 g and resuspended in glycerol buffer (30% glycerol, 150 mM NaCl, 25 mM TrisHCl, 1 mM MgCl_2_, pH 7.5). Protein concentrations were measured by Bradford assay (BioRad, Hercules, CA, USA) and 10–20 µg of protein were separated on a SDS-7.5% PAGE for PBP2a Western blots and 80 µg of protein were separated on a SDS-12% PAGE for PBP4 Western blots. Gels were blotted onto PVDF-membranes (Immobilon-P, Milipore, Billerica, MA, USA) and the membranes were blocked with 5% milk powder and with 40 µg/ml human IgG (Calbiochem, Merck, Darmstadt, Germany). PBP2a was detected using PBP2a antibodies (1∶20’000, Denka Seikeņ Tokyo, Japan) and Horseradish Peroxidase-Conjugated (HRP) goat anti-mouse IgG (1∶2500, Jackson ImmunoResearch, West Grove, PA, USA). PBP4 was detected with PBP4 antibodies (1∶2000, [50]) and goat anti-rabbit IgG HPR (1∶5000, Jackson ImmunoResearch). Signals were quantified using the AlphaInnotech imager software (Santa Clara, CA, USA), loading differences were corrected and values were given as a percentage of the signal intensity of strain RA120 with standard deviation. Student’s *t*-test was applied to determine the significance of any differences. Western blot analyses were performed on three independent protein extractions.

### Bocillin-FL staining of PBPs

Cell membrane fractions were extracted as described above. Eighty µg of proteins from membrane fractions were incubated for 30 min at 35°C with the fluorescent penicillin analogue Bocillin-FL (Invitrogen) as previously described [51]. Samples were separated by a SDS-7.5% PAGE and fluorescence was visualized with the FluorChem™ SP imaging system (AlphaInnotech).

### NaCl tolerance

Overnight cultures of strains were transferred into a single row of a microtiter plate and serial 5-fold dilutions in 0.85% NaCl were prepared. Dilutions were replica-plated onto increasing NaCl concentrations using an automatic inoculator (Microtiter, Dynatech AG, Switzerland). All plates were incubated at 37°C for 48 h and three independent experiments were performed.

### Luciferase assays

Luciferase measurements were performed as described earlier [19]. Briefly, cultures were inoculated at OD 0.05 and 1 ml samples were harvested by centrifugation after 1.5, 3, 4.5, 6 and 7.5 hours. Pellets were thawed and resuspended in PBS to OD 10. Cell suspensions were mixed with equal amounts of Luciferase Assay System substrate (Promega, Madison, WI, USA) and luminescence was measured on a Turner Designs TD-20/20 luminometer (Promega). For statistical analysis of individual time points and for comparison of whole data sets, Student’s *t*-test and two-way Anova were applied, respectively.

### Cell sampling for c-di-AMP quantification

Overnight cultures were diluted to OD 0.05 and grown to OD 2. Cells were harvested by filtration using 0.2 µm RC filters (Sartorius Stedium Biotech, Göttingen, Germany) collecting 3 ml culture per filter and two filters for each of the five replicates. Filters were washed once with 10 ml Braun water (37°C, B. Braun Meslungen AG, Meslungen, Germany) and directly transferred into 12 ml of pre-cooled quenching solution (–20°C, 60% acetonitril (Sigma, CHROMASOLV® Plus), 20% methanol (Sigma), 20% 0.5 M formic acid). Three hundred µl of 0.5 µM c-di-GMP (Biolog, Bremen, Germany) solution were added as an internal standard and the solution was sonicated four times for 20 seconds before it was snap frozen in liquid nitrogen and lyophilised.

### c-di-AMP analysis by LC-MS

Analyses were performed with a Rheos 2200 HPLC system (Flux Instruments, Basel, Switzerland) coupled to an LTQ Orbitrap mass spectrometer (Thermo Fisher Scientific, Waltham, MA, USA), equipped with an electrospray ionisation probe. c-di-AMP esters were analyzed by a method previously used for online solid phase extraction (SPE) and LC-MS analysis of CoA esters [52] with the exception of the MS analysis which was optimized for ionization of c-di-AMP (for parameters see below). Besides desalting, the method allowed complete removal of polar compounds including mono-nucleotides from cell extracts and samples could be injected at higher concentration. To perform online SPE of c-di-AMP and c-di-GMP prior to LC separation two C18 analytical columns (Gemini 50×2.0 mm and 100×2.0 mm, particle size 3 µm; Phenomenex, Torrance, CA, USA) were used. Flow rate was 220 µl/min, solvent A was 50 mM formic acid adjusted to pH 8.1 with NH_4_OH and solvent B was methanol. The injection volume was 10 µl. For online desalting, samples were loaded on 50×2.0 mm C18 column and the sample was washed on column for 5 min with 100% solvent A. During SPE the short column was connected to waste via a 6-port-valve and the 100×2.0 mm column was equilibrated with solvent A by an additional pump. After desalting both columns were connected in series and the following gradient of B was applied to separate c-di-AMP: 5 min, 5%; 15 min, 23%; 25 min, 80%; 27 min, 80%. The LC-MS system was equilibrated for 6 min at initial elution conditions between two successive analyses. The LC was coupled to the mass spectrometer. Sheath gas flow rate was 40, auxiliary gas flow rate was 30, tube lens was –93 V, capillary voltage was –35 V, and ion spray voltage was –4.0 kV. MS analysis was done in the negative FTMS mode at a resolution of 60,000 (m/z 400).

### Quantification of c-di-AMP

c-di-AMP was quantified using c-di-GMP as an internal standard to compensate for loss during sample preparation. Though c-di-GMP and c-di-AMP are chemically very similar, they do not co-elute and other compounds present in the sample can influence electrospray ionization of compounds. Therefore, the area response factors of the two compounds were determined in a series of standard solutions relative to the series of *S. aureus* cell extracts spiked with standards. The slope of a linear calibration curve (y = m*x+b) was determined for both compounds in standard and in cell extracts and slope ratios (m_sample_/m_standard_) were calculated. Values were used to correct corresponding peak areas measured in *S. aureus* extracts. The detection limit of 32 nM c-di-AMP (corresponding to 0.3 pmol c-di-AMP on the column) was estimated from the intercept of the calibration curve.

## Results and Discussion

### Identification of mutation in the diadenylate cyclase gene *dacA*

In previous studies a highly and homogeneously resistant MRSA was constructed by introducing the SCC*mec* element from strain COL into the methicillin sensitive *S. aureus* (MSSA) strain BB255 [53], [54]. Introduction of the resistance cassette caused a fitness cost and significantly reduced the growth rate of the resulting MRSA strain RA120. From this slow growing strain RA120, a faster growing variant, ME51, was isolated during a competitive growth experiment [54]. Rescue from the fitness burden was accompanied by reduced, heterogeneous resistance to oxacillin [54]; a phenotype observed in CA-MRSA strains [4]. Genome sequencing of these two MRSA strains, RA120 and ME51, revealed 80 SNPs or deletion/insertion polymorphisms (DIPs) present in both strains, compared to the published NCTC8325 sequence (GenBank accession CP000253). Therefore, the parent strain BB255 was subsequently sequenced, confirming that these SNPs were already present in BB255, which excluded the possibility that these genetic differences were connected to the introduction of the SCC*mec* element (complete Table S1). All 46 sequencing errors identified in the NCTC8325 sequence by Berscheid *et al*. were confirmed in BB255 [55]. Twelve SNPs and one DIP were likely to be additional sequencing errors in the published NCTC8325 sequence since they were present in all other published *S. aureus* genomes including the NCTC8325 derivative RN4220 [56] (Table 2, marked with a cross in column RN4220). Thus, 21 SNPs or DIPs were identified to be real differences between BB255 and other NCTC8325 derivatives (Table 2). These mutations could either have been naturally acquired over time or caused by the ethidium bromide treatment of the original NCTC8325 FK268 that was used to cure it from its plasmid to create strain BB255 [53].

**Table 2:** SNPs and DIPs identified in BB255, RA120 and ME51.

Position^1^	Ref^2^	Seq^3^	Impact	Locus (SAOUHSC_)^1^	Description^4^	RN4220^5^
5286	G	A	Gly85Ser	00166	DNA gyrase, ATP hydrolyzing subunite B	
22181	C	A		Intergenic 00018/00019	Non-coding	x
47652	T	-		Intergenic 00044/00045	Non-coding	x
73564	G	T	Asn472Lys	00069	Partial *spa* gene for immunglobulin G binding protein A	
142255	G	T	Gly200Trp	00136	CHP, nitrate transport ATP-binding protein NrtD, Putative ABC transporter	
210632	T	A	Asp454Glu	00190	CHP, membrane domain of membrane-anchored glycerophosphoryl diester phosphodiesterase	
218932	C	T	Asp63Asn	00197	Putative acyl-CoA dehydrogenase domain protein	
230630	T	G	Silent	00209	Putative PTS system maltose-and glucose-specific EIICB component	
252494	G	A	Gly323Asp	00230	Two-component sensor histidine kinase LytS	
329229	T	C	Leu131Ser	00314	Possible transcriptional regulator MarR family, MATE family multi-antimicrobial extrusion protein	
433209	A	T		Intergenic 00434/00435	Non-coding	
523591	G	A	Glu431Lys	00524	DNA-directed RNA polymerase beta subunit	
541723	T	G		Intergenic 00535/00536	Non-coding	
541724	G	C		Intergenic 00535/00536	Non-coding	
649126	G	T	Silent	00661	CHP, putative lipase/esterase	x
841103	G	T	Silent	00877	Iron-sulphur cluster assembly accessory protein	x
841139	G	T	Silent	00877	Iron-sulphur cluster assembly accessory protein	x
980692	C	T	Pro301Leu	01009	Phosphoribosylaminoimi-dazole carboxylase, ATPase subunit	
1013608	G	A	Val50Met	01044	CHP, putative transcriptional regulator	
1653482	G	A	Silent	01748	tRNA-guanine transglycosylase	x
2243145	G	–		R0005	rRNA-16S Ribosomal RNA	
2243146	G	–		R0005	rRNA-16S Ribosomal RNA	
2318272	G	A		Intergenic 002512/02515	Non-coding	x
2318274	G	T		Intergenic 002512/02515	Non-coding	x
2318290	C	A		Intergenic 002512/02515	Non-coding	x
2331612	C	A	Ala30Ser	02527	Peptidoglycan pentaglycine interpeptide biosynthetic protein FmhB (FemX)	
2383630	G	T	Silent	02591	CHP, putative membrane protein	x
2383660	G	T	Silent	02591	CHP, putative membrane protein	x
2466536	T	C	Lys349Glu	02681	Nitrate reductase, alpha subunit	
2556234	A	G		Intergenic 02781/02782	Non-coding	
2596878	C	T	Gly107Asp	02818	MFS family major facilitator transporter	
2678563	T	C	Silent	02911	CHP	x
2684051	C	T	Ala90Thr	02919	3-methyl-2-oxobutanoate hydroxymethyltransferase	
2689048	G	T	Val353Leu	02923	Amino acid permease	x

1 Genome positions and locus numbers are according to NCTC8325 sequence (GenBank accession CP000253). ^2^Reference nucleotide in NCTC8325. ^3^Sequenced nucleotide in BB255, RA120 and ME51 determined in this study. ^4^Description of putative gene products and functions were taken from NCTC8325 annotations and were improved from annotations of other *S. aureus* strains. ^5^x in this column indicates SNP/DIP is present in RN4220 [56]. Abbreviation: CHP, conserved hypothetical protein.

The SCC*mec* element introduced from strain COL was identical in both strains RA120 and ME51, but had four SNPs and a deletion when assembled against the published SCC*mec* sequence of COL (GenBank accession CP000046). One mutation was found in a glycerophosphoryl diester phosphodiesterase (COL genome position G38611A, SACOL_0031, Asp95Asn) and one in a conserved hypothetical protein (C51476T, SACOL_0044, Cys42Tyr). In the gene of the methicillin resistance surface protein Pls two silent SNPs (G57595A, A61354T) and a five nucleotide deletion at position 61363-61367, leading to the loss of amino acids and a frame shift resulting in truncation of Pls at amino acid 1383, were detected.

In addition to the SNPs and DIPs present in both strains, the genome sequences of RA120 and ME51 differed by only one nucleotide in the recently discovered c-di-AMP cyclase gene *dacA (*SAOUHSC_02407*)* [29], [33], which was confirmed by Sanger sequencing. The SNP was identified in ME51, resulting in the change of a highly conserved glycine to a serine (Gly206Ser, genome position C2235346T in NCTC8325) which is separated by only one amino acid from the proposed functional motive RHR of the diadenylate cyclase [33].

### Reconstruction of the *dacA-*SNP

**Figure 1:**
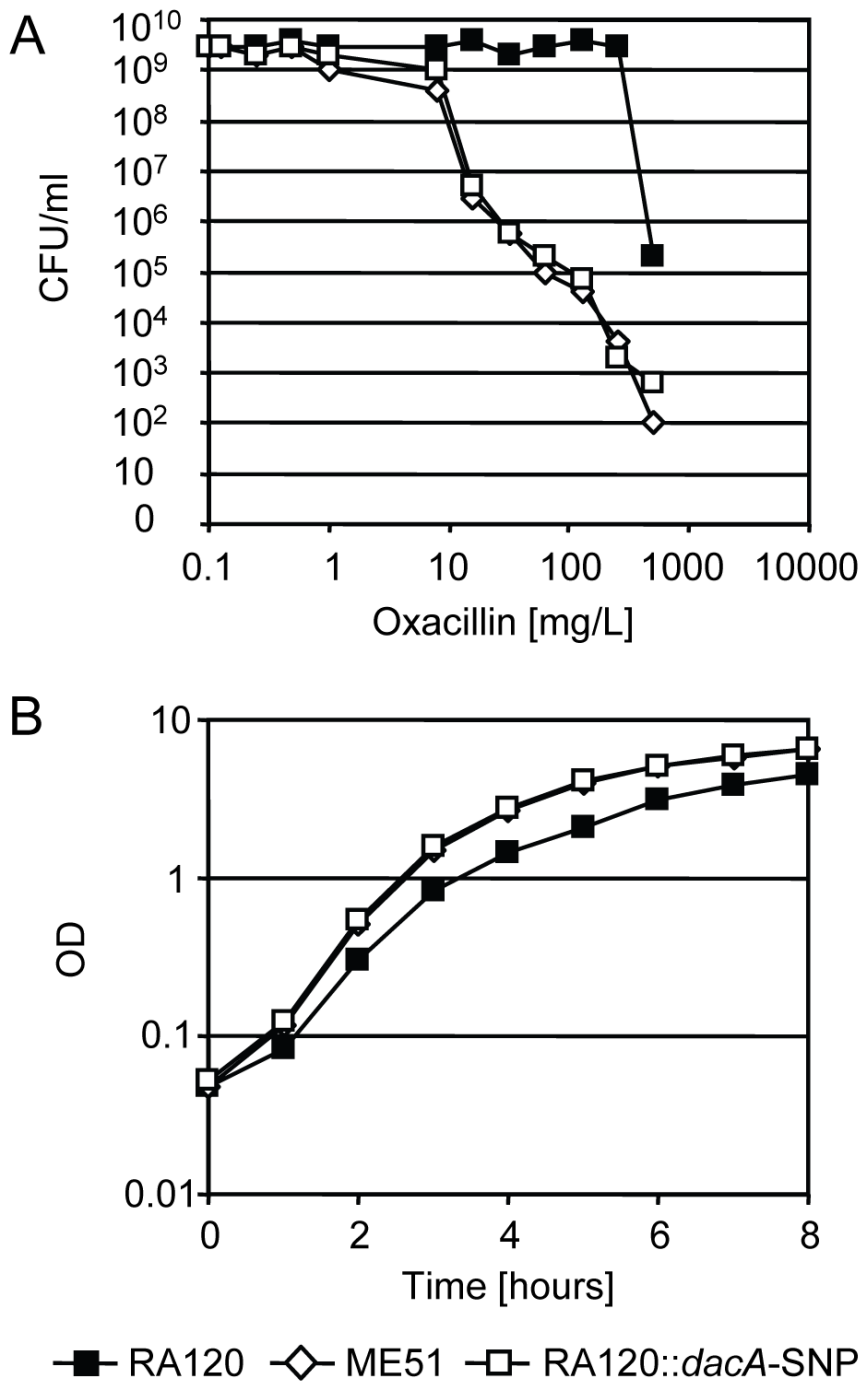
Oxacillin resistance and fitness of RA120, ME51 and RA120::*dacA*-SNP. A, oxacillin population analysis profiles and B, growth curves of the strain RA120 (black squares) and the *dacA*-SNP strains ME51 (white diamonds) and RA120::*dacA*-SNP (white squares). Representative data from three independent experiments are shown.

To confirm a direct connection between the mutation in the *dacA* gene and the phenotypic changes in ME51 that resulted in it becoming a fitter but heterogeneously resistant strain, the mutation was artificially introduced into the homogeneously high-level resistant but slow growing strain RA120 by homologous recombination, resulting in strain RA120::*dacA-*SNP. Upon introduction of the SNP into RA120, its resistance profile became heterogeneous (Figure 1A) and the oxacillin MIC decreased from >256 mg/L for RA120 to 64 mg/L for RA120::*dacA*-SNP. Like ME51, strain RA120::*dacA*-SNP grew faster than RA120 (Figure 1B). The growth rate increased significantly (p<0.01) upon introduction of the *dacA*-SNP with a reduction of the doubling time from 39.5±1.7 min for RA120 to 29. 8±1.6 min for RA120::*dacA*-SNP in the exponential growth phase. These resistance and fitness features of RA120::*dacA*-SNP were comparable to ME51 which had a doubling time of 29.0±2.1 and an oxacillin MIC of 64 mg/L. Several recently published studies found that mutations in the c-di-AMP phosphodiesterase *gdpP* increased resistance to beta-lactam antibiotics [38], [39], [40], [41]. The study of Corrigan *et al*. quantified the c-di-AMP levels in a *gdpP* mutant confirming thereby that elevated c-di-AMP levels are associated with increased beta-lactam resistance [29]. We hypothesized that the opposite occurred in ME51. The amino acid substitution close to one of the functional motifs in DacA could reduce the efficiency of the cyclase resulting in a lower c-di-AMP level and decreased beta-lactam resistance. The growth rate of the constructed mutant RA120::*dacA*-SNP increased to a level that was comparable to that of the naturally selected mutant ME51 (Figure 1B), suggesting a direct connection between increased fitness and the mutation in *dacA*.

### Quantification of c-di-AMP and penicillin binding proteins (PBPs)

**Figure 2:**
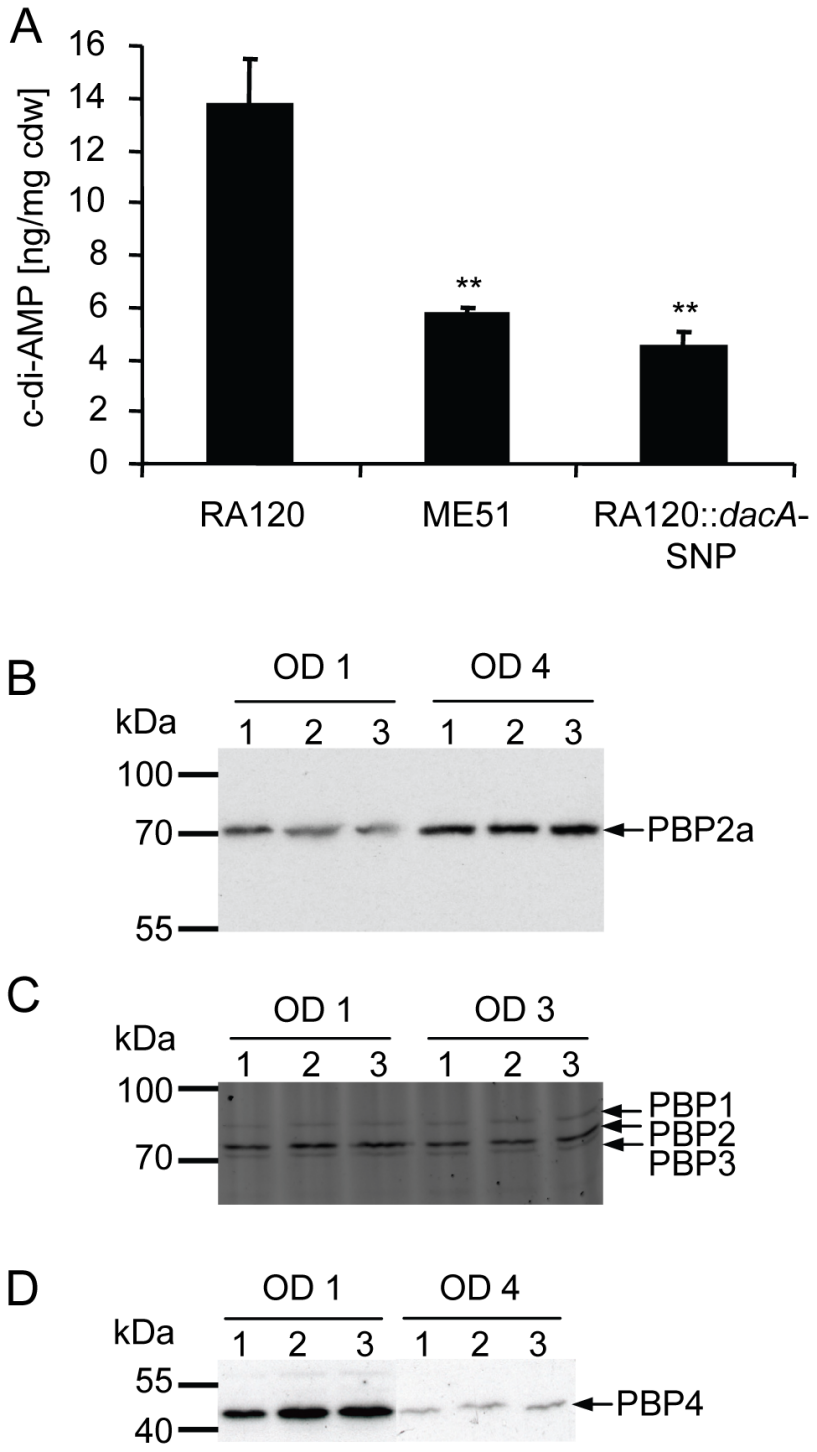
Cellular levels of c-di-AMP and PBPs of RA120, ME51 and RA120::*dacA*-SNP. A, cellular levels of c-di-AMP quantified by LC-MS in ng per mg cellular dry weight (cdw). Average values from five biological replicates with standard deviation are shown. Asterisks indicate significant differences compared to RA120 (** p<0.01). B, PBP2a (76 kDa) Western blot analysis, C, visualisation of PBP1 (83 kDa), PBP2 (80 kDa) and PBP3 (77 kDa) using the fluorescent penicillin analogue Bocillin-FL and D, PBP4 (48 kDa) Western blot analysis of membrane fractions of the strains RA120 (1), ME51 (2) and RA120::*dacA*-SNP (3) sampled at indicated ODs. Representative results from three independent protein extractions are shown.

To prove our hypothesis that the *dacA* mutation reduces the cellular level of c-di-AMP, LC-MS analysis of RA120, ME51 and RA120::*dacA*-SNP were performed to quantify c-di-AMP. The amount of c-di-AMP per mg cellular dry weight (cdw) was 13.8±1.7 ng/mg cdw for the wild type strain RA120 and significantly reduced to 5.8±0.2 ng/mg cdw and 4.5±0.5 ng/mg cdw for ME51 and RA120::*dacA*-SNP, respectively (Figure 2A). Compared to the up to 15-fold increase seen in c-di-AMP upon *gdpP* mutation [29] this is a rather small decrease which, however, had a pronounced effect on fitness and resistance.

Pozzi and co-workers showed that a heterogeneously resistant strain was transformed into a homogeneously resistant strain that produced increased amounts of PBP2a, upon mutation in the c-di-AMP phosphodiesterase *gdpP* [41]. Thus, one explanation for a reduction of resistance in the strains ME51 and RA120::*dacA*-SNP could be a decreased level of PBP2a. However, in our strains no significant difference in PBP2a production was detected upon *dacA* mutation neither in ME51 nor in RA120::*dacA*-SNP (Figure 2B). Quantification of signal intensities from PBP2a Western blots gave values of 96±6% for ME51 at OD1 and 104±5% at OD 4, relative to the signal intensity of RA120. Similar values were detected for RA120:*:dacA*-SNP resulting in intensity percentages of 98±4% at OD 1 and 106±6% at OD 4, compared to RA120. In the study of Pozzi *et al.* the effect of their *gdpP* mutation on the c-di-AMP level was not quantified, but it may be assumed from the studies of Corrigan *et al*. that *gdpP* mutations have a more drastic effect on c-di-AMP levels than the *dacA*-SNP identified in this study. This could be reflected in the magnitude of PBP2a level changes [29], and explain why we could not detect a difference in PBP2a levels in our *dacA*-SNP mutants. Alternatively, the *dacA*-SNP may influence methicillin resistance independently of the amount of PBP2a. c-di-AMP was previously found to affect cell wall homeostasis and elevated levels of c-di-AMP increased peptidoglycan cross linking by an as yet unknown mechanism [29], [40]. These findings, together with the fact that *gdpP* mutations in MSSA strain backgrounds lead to increased beta-lactam MICs [39] and our observation of decreased oxacillin resistance in MSSA strain BB255 upon *dacA*-SNP introduction (see below), suggest that the effects of c-di-AMP on beta-lactam resistance are likely to be at least partially independent of PBP2a levels. However, more research is required to understand the mechanism by which c-di-AMP affects cell wall homeostasis and beta-lactam resistance.

Since PBP2 and PBP4 were previously shown to affect beta-lactam resistance, the protein levels of endogenous PBPs were also analysed [15], [16], [57], [58]. Qualitative analysis of the amount of the endogenous PBPs PBP1, PBP2 and PBP3 using the fluorescent penicillin analogue Bocillin-FL did not reveal any obvious differences between the strains RA120, ME51 and RA120::*dacA*-SNP (Figure 2C). Interestingly, PBP4 Western blots showed seemingly higher amounts of PBP4 in *dacA*-SNP strains ME51 and RA120::*dacA*-SNP, which, however, was not significantly different from RA120 due to large variations between experiments (Figure 2D). Quantification of Western blot signals as in percentages of the signal intensity of RA120 gave values of 197±61% and 181±62% at OD 1, and 171±52% and 190±58% at OD 4, respectively. This observation was unexpected, since previous studies had shown that PBP4 levels directly correlated with beta-lactam resistance; overexpression of PBP4 leading to increased beta-lactam resistance [57] and deletion of PBP4 reducing resistance levels [16], [58], [59]. Even though the importance of PBP4 for beta-lactam resistance was shown to be very strain dependent [16], we would not have expected to see higher PBP4 levels in the *dacA*-SNP strains that had reduced oxacillin MICs. However, it is possible that despite increased total amounts, PBP4 activity or localisation could be impaired and further research is required to evaluate if there is a connection between c-di-AMP levels and PBP4 levels, activity or localisation.

### Cell envelope associated phenotypes of *dacA*-SNP

**Figure 3:**
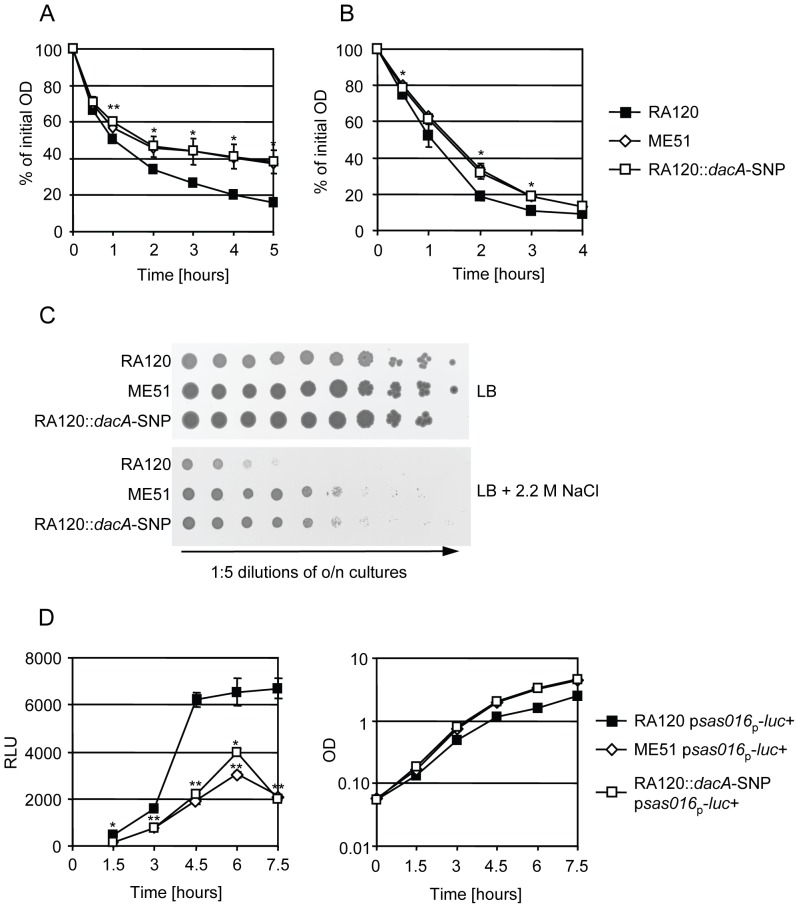
Analysis of phenotypes associated with cell envelope properties in RA120, ME51 and RA120::*dacA*-SNP. A, spontaneous autolysis of RA120 (black squares) and the *dacA*-SNP strains ME51 (white diamonds) and RA120::*dacA*-SNP (white squares) in phosphate buffer and B, induced autolysis of strains with 0.01% Triton X-100 in phosphate buffer. Average values with standard deviation from three independent experiments are shown. Asterisks indicate measurement time points with significant difference between both RA120/ME51 and RA120/RA120::*dacA*-SNP (* p<0.05, ** p<0.01) using Student’s *t*-test. Two-way ANOVA analysis considering all time points resulted in significant differences (p<0.01) for stain pairs RA120/ME51 and RA120/RA120::*dacA*-SNP for both spontaneous and induced autolysis. C, NaCl tolerance of RA120, ME51 and RA120::*dacA*-SNP. Overnight cultures were serially diluted 1∶5 and plated on plain LB agar or LB agar with 2.2 M NaCl. Representative results from three independent experiments are shown. D, left, relative light units (RLU) measured from cell wall stress stimulon reporter construct p*sas016*_p_ *-luc+* in RA120 (black squares) and the *dacA*-SNP strains ME51 (white diamonds) and RA120::*dacA*-SNP (white squares) and right, the corresponding OD values of the cultures at each sampling point. Average values with standard deviation from three independent experiments are shown. Asterisks indicate measurement time points with significant differences in RLU between both RA120/ME51 and RA120/RA120::*dacA*-SNP strain pairs. If the strain pairs had different categories of p-values these were indicated separately (* p<0.05, ** p<0.01). Two-way ANOVA analysis considering RLU values from all time points resulted in significant differences (p<0.01) for both strain pairs RA120/ME51 and RA120/RA120::*dacA*-SNP.

Additional effects of this more than 2-fold decrease in the cellular c-di-AMP level were further investigated by phenotypic characterisation of RA120 and the *dacA*-SNP strains ME51 and RA120::*dacA*-SNP. In *S. aureus*, elevated c-di-AMP levels caused by *gdpP* mutations were found to affect cell envelope homeostasis resulting in increased autolysis and increased resistance to lysostaphin and oxacillin [29]. As expected, we observed the opposite effect in *dacA*-SNP strains. The *dacA*-SNP clearly decreased resistance to oxacillin as shown above (Figure 1A) and decreased both spontaneous and Triton X-100 induced autolysis (Figure 3AB). However, resistance to lysostaphin was not affected (data not shown).

A study in *Lactococcus lactis* revealed salt hypersensitivity in a spontaneous *gdpP* mutant [45]. Although, *S. aureus* can tolerate high salt concentrations [60], we could detect decreased salt sensitivity in the mutants with lower c-di-AMP levels (Figure 3C). Inversely, increased salt sensitivity was observed very recently for a *S. aureus gdpP* mutant with 15-fold increased c-di-AMP levels and for a mutant of the newly identified c-di-AMP target *ktrA*, a potassium transporter-gating component [46].

Since previous studies suggest c-di-AMP to be involved in cell envelope homeostasis and cell envelope stress [29], [40] we analysed the effect of the *dacA*-SNP on expression of the cell wall stress stimulon (CWSS). Induction of the CWSS, a set of genes controlled by the two component system VraSR, is a good indicator of cell envelope stress caused by disturbance of cell envelope biosynthesis [19], [28], [61]. CWSS expression is up-regulated by depletion or deletion of cell envelope biosynthesis enzymes or upon exposure to cell wall-targeting antibiotics [19], [61], [62], [63], [64], [65], [66]. Using the well established reporter plasmid p*sas016*_p_ *-luc*+ which contains the promoter of *sas016*, a highly responsive CWSS gene of unknown function, fused to a luciferase gene [65], [67], the CWSS expression was measured indirectly in relative light units (RLU). Indeed, we detected a lower basal CWSS expression over growth in the *dacA*-SNP strains ME51 and RA120::*dacA*-SNP (Figure 3D). The expression level in the parent strain RA120 was about 3-fold higher than in the *dacA*-SNP strains. These findings suggest that cell wall stress stimulon expression could correlate directly with cellular c-di-AMP levels.

### Effect of the *dacA*-SNP on fitness, resistance and c-di-AMP levels in an MSSA strain and in an MRSA of different genetic background

**Figure 4:**
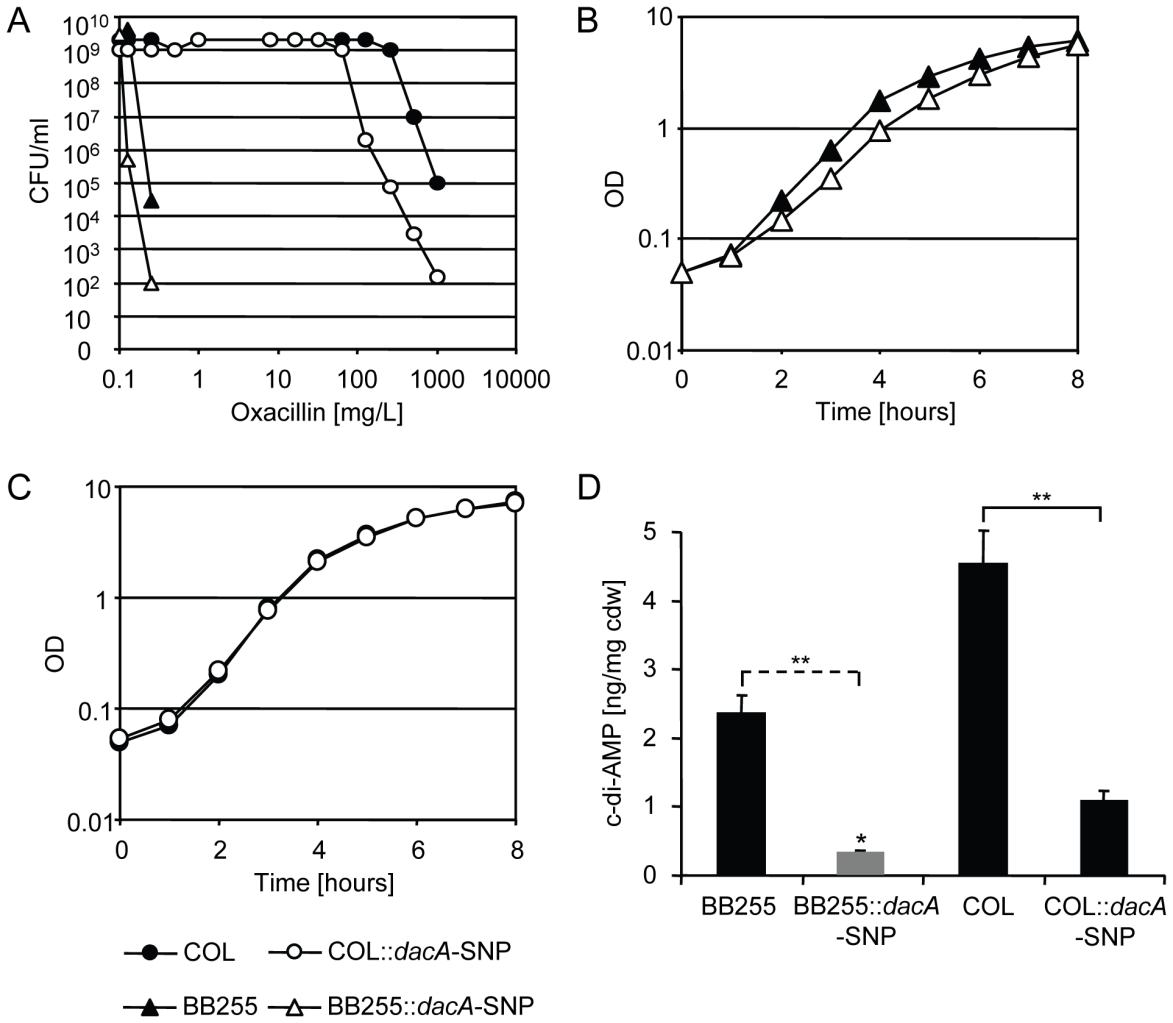
Effect of *dacA*-SNP on oxacillin resistance, fitness and the c-di-AMP levels in MSSA strain BB255 and MRSA strain COL. A, oxacillin population analysis profile of wild type strains BB255 (black triangle) and COL (black circle) and their corresponding *dacA*-SNP strains BB255::*dacA*-SNP (white triangle) and COL::*dacA-*SNP (white circle). Representative data from three independent experiments are shown. B, growth curves of BB255 and BB255::*dacA*-SNP and C, growth curves of COL and COL::*dacA*-SNP. Representative data from three independent experiments are shown. D, cellular levels of c-di-AMP quantified by LC-MS in ng per mg cellular dry weight (cdw). Average values from five biological replicates with standard deviation are shown. Grey bar with asterisk (*) indicates c-di-AMP concentration was blow detection limit and the given value represents maximal concentration which was estimated by dividing the limit of detection (32 nM) by the samples biomass concentrations. Two asterisks (**) indicate a significant difference using Student’s *t*-test (p<0.01) and the dashed bracket indicates statistical analysis was performed using the estimated maximal value for BB255::*dacA*-SNP.

To determine the general importance of DacA in different strain backgrounds, the *dacA*-SNP was introduced into the MSSA parent strain BB255 and into the MRSA strain COL which both carried *dacA* genes that were 100% identical to *dacA* in RA120. Both resulting mutants showed increased oxacillin susceptibility (Figure 4A). The number of surviving cells decreased in the methicillin sensitive strain BB255::*dacA*-SNP by approximately 1000-fold on plates containing 0.125 mg/L or 0.25 mg/L of oxacillin, which was also reflected by a reduction of the oxacillin MIC from 0.25 mg/L in BB255 to 0.125 mg/L in BB255::*dacA*-SNP. In the COL::*dacA*-SNP mutant a lowered and heterogeneous resistance profile was observed, though the effect was slightly less pronounced than in the BB255 derived MRSA RA120::*dacA*-SNP. The oxacillin MIC for COL decreased from >256 mg/L to 256 mg/L upon introduction of *dacA*-SNP.

The impact of the *dacA*-SNP on fitness varied between the different strains. The MSSA strain BB255 showed a decrease in the growth rate upon the introduction of the *dacA*-SNP (Figure 4B); the doubling time increased from 30±2.1 min for BB255 to 42±3.2 min for BB255::*dacA*-SNP. In COL, the SNP did not significantly affect the minimal doubling time of the strain with 38. 2±1.1 min and 40.0±1.9 min for COL and for COL::*dacA*-SNP, respectively (Figure 4C). Quantification of c-di-AMP once again showed clear reductions in cellular c-di-AMP levels upon *dacA*-SNP introduction into both backgrounds (Figure 4D).Introduction of the *dacA*-SNP into MRSA COL reduced the c-di-AMP level about four-fold, from 4.5±0.5 to 1.1±0.1 ng/mg cdw. The induction of *dacA*-SNP into BB255 reduced the c-di-AMP of 2.4±0.3 ng/mg cdw to a level below the limit of detection. The value included in the graph as a grey bar corresponds to the limit of detection (32 nM) divided by samples biomass concentrations. The reduction to a very low c-di-AMP level might be an explanation for the reduced growth rate of BB255::*dacA*-SNP, since c-di-AMP was claimed to be essential for *S. aureus* [29] and reduced c-di-AMP levels were found be detrimental for growth of *B. subtilis* [34] and *L. monocytogenes* [42].

Surprisingly, the c-di-AMP level in BB255, the RA120 parent strain, was about 6-fold lower than the c-di-AMP level in RA120. Therefore, it could be speculated that introduction of the SCC*mec* element into BB255 led to non genetic adaptations of yet unknown nature, resulting in an elevated c-di-AMP level in RA120, which was reduced by the *dacA* mutation to a wild type level in ME51 and RA120::*dacA*-SNP. However, to our knowledge, no studies on the natural variation of the c-di-AMP levels in different strain backgrounds have been performed yet and current data suggests that c-di-AMP levels are not generally higher in MRSA, since Corrigan *et al.* did not detect a difference in c-di-AMP levels between MRSA and MSSA [29]. Thus, more work is required to confirm and explain the possibly elevated c-di-AMP levels in RA120 and to generally understand the mechanisms by which c-di-AMP affects beta-lactam resistance. So far, forces driving acquisition of mutations in c-di-AMP synthesising gene *dacA* or the degrading enzyme *gdpP* seem to include stress caused by i) selective pressure by beta-lactams [38], [39], [41] ii) disturbance of cell envelope integrity due to LTA-deficiency [29] or iii) selective pressure on fitness such as the conditions occurring in mixed growth competition experiments ([54], this work). Why and how altering c-di-AMP levels under these conditions might be an advantage for *S. aureus* remains to be elucidated. As a second messenger, c-di-AMP can be expected to control several factors involved in different cellular processes, which might explain why alterations in c-di-AMP levels can lead to such diverse phenotypes as changes in antibiotic resistance, cell envelope stability, cell division, biofilm formation, growth rate, transcription of regulators, expression of virulence factors and pathogenicity.

## Conclusion

This is the first time that a mutation in *dacA*, resulting in decreased c-di-AMP levels, was shown to reduce methicillin resistance and increase growth rates. Markerless allelic replacement confirmed that the *dac*A mutation could transform a highly homogenously methicillin resistant strain into a faster growing but lower-level and heterogeneously resistant MRSA. The impact of the *dacA*-SNP on c-di-AMP levels and fitness seemed to vary between strain backgrounds. Analysis of cell envelope properties of the strains with the *dacA* mutation revealed reduced autolysis, increased salt tolerance and a reduction in the basal expression level of the cell wall stress stimulon, providing further confirmation for a cell envelope associated signalling function of c-di-AMP. Further research is required to understand how cellular c-di-AMP levels influence fitness and resistance. This will involve investigating possible connections between the phenotypes associated with altered c-di-AMP levels and the five recently identified c-di-AMP targets and the potential identification of additional targets of c-di-AMP-dependent regulation.

## Supporting Information

Table S1 **Complete list of differences in BB255, ME51 and RA120 compared to the NCTC8325 sequence including SNPs and DIPs identified by Berscheid et al. 2012** [**55**] **.**
(DOC)
