# Supplementary material for: Mutation in the C-Di-AMP Cyclase dacA Affects Fitness and Resistance of Methicillin Resistant Staphylococcus aureus
Source: PLoS One. 2013 Aug 27;8(8):e73512. doi: 10.1371/journal.pone.0073512 (PMC3754961; doi:10.1371/journal.pone.0073512)
Supplement: Table S1 — Complete list of differences in BB255, ME51 and RA120 compared to the NCTC8325 sequence including SNPs and DIPs identified by Berscheid et al. 2012 [55] . (DOC) [file pone.0073512.s001.doc]

**Table S1:** Complete list of differences in BB255, ME51 and RA120 compared to the NCTC8325 sequence including SNPs and DIPs identified by Berscheid et al. 2012 [55].

| **Position**^1^ | **Ref**^2^ | **Seq**^3^ | **Impact** | **Locus (SAOUHSC_)**^1^ | **Description**^4^ |
| --- | --- | --- | --- | --- | --- |
| 5286 | G | A | Gly85Ser | 00166 | DNA gyrase, ATP hydrolyzing subunite B |
| 22181 | C | A |  | Intergenic 00018/00019 | Non-coding |
| 47652 | T | - |  | Intergenic 00044/00045 | Non-coding |
| 73564 | G | T | Asn472Lys | 00069 | Partial *spa* gene for immunglobulin G binding protein A |
| 142255 | G | T | Gly200Trp | 00136 | CHP, nitrate transport ATP-binding protein NrtD, putative ABC transporter |
| 210632 | T | A | Asp454Glu | 00190 | CHP, membrane domain of membrane-anchored glycerophosphoryl diester phosphodiesterase |
| 218932 | C | T | Asp63Asn | 00197 | Putative acyl-CoA dehydrogenase domain protein |
| 230630 | T | G | Silent | 00209 | Putative PTS system maltose-and glucose-specific EIICB component |
| 252494 | G | A | Gly323Asp | 00230 | Two-component sensor histidine kinase LytS |
| 329229 | T | C | Leu131Ser | 00314 | Possible transcriptional regulator MarR family, MATE family multi-antimicrobial extrusion protein |
| 433209 | A | T |  | Intergenic 00434/00435 | Non-coding |
| 523591 | G | A | Glu431Lys | 00524 | DNA-directed RNA polymerase beta subunit |
| 541723 | T | G |  | Intergenic 00535/00536 | Non-coding |
| 541724 | G | C |  | Intergenic 00535/00536 | Non-coding |
| 590402 | G | – | Frame shift | 00591 | CHP |
| 649126 | G | T | Silent | 00661 | CHP, putative lipase/esterase |
| 841103 | G | T | Silent | 00877 | Iron-sulphur cluster assembly accessory protein |
| 841139 | G | T | Silent | 00877 | Iron-sulphur cluster assembly accessory protein |
| 947898 | C | – | Frame shift | 00973 | Putative glycosyl transferase |
| 980692 | C | T | Pro301Leu | 01009 | Phosphoribosylaminoimidazole carboxylase, ATPase subunit |
| 1013608 | G | A | Val50Met | 01044 | CHP, putative transcriptional regulator |
| 1042000 | T | – | Frame shift | 01078 | 50S ribosomal protein L32, *rpmF* |
| 1160513 | G | A | Ala106Thr | 01209 | 16S rRNA processing protein RimM |
| 1160531 | A | G | Lys112Glu | 01209 | 16S rRNA processing protein RimM |
| 1180886 | G | – | Frame shift | 01232 | 30S ribosomal protein S2, *rpsB* |
| 1283784 | C | – | Frame shift | 01342 | Exonuclease SbcC |
| 1562913 | A | T | Stop337Lys | 01649 | Peptidase, rhomboid family protein |
| 1632635 | – | A | Frame shift | 01726 | Putative tRNA methyltransferase MnmA |
| 1636251 | T | – | Frame shift | 01732 | BadM/Rrf2 family transcriptional regulator |
| 1653482 | G | A | Silent | 01748 | tRNA-guanine transglycosylase |
| 1683491 | T | C | Lys40Glu | 01786 | Translation initiation factor IF-3 (InfC) |
| 1733515 | G | T | Thr73Asn | 01827 | Septation ring formation regulator EzrA |
| 1733572 | A | G | Phe54Ser | 01827 | Septation ring formation regulator EzrA |
| 1981053 | A | G | Phe92Ser | 02107 | Putative UDP-N-acetylmuramyl-tripeptide synthetase |
| 2087725 | A | T | Phe218Ile | 02254 | Chaperonin GroEL |
| 2166163 | G | C | Thr124Arg | 02337 | UDP-N-acetylglucosamine  1-carboxyvinyltransferase MurA |
| 2166183 | C | A | Silent | 02337 | UDP-N-acetylglucosamine  1-carboxyvinyltransferase MurA |
| 2221850 | C | A | Ser244Tyr | 02401 | Putative transcriptional anti-terminator |
| 2243145 | G | – |  | R0005 | rRNA-16S ribosomal RNA |
| 2243146 | G | – |  | R0005 | rRNA-16S ribosomal RNA |
| 2244932 | C | – | Frame shift | 02417 | Putative ATP-binding protein, Mrp/Nbp35 family |
| 2244933 | T | – | Frame shift | 02417 | Putative ATP-binding protein, Mrp/Nbp35 family |
| 2296654 | G | – | Frame shift | 02474 | CHP |
| 2318272 | G | A |  | Intergenic 002512/02515 | Non-coding |
| 2318274 | G | T |  | Intergenic 002512/02515 | Non-coding |
| 2318290 | C | A |  | Intergenic 002512/02515 | Non-coding |
| 2331612 | C | A | Ala30Ser | 02527 | Peptidoglycan pentaglycine interpeptide biosynthetic protein FmhB (FemX) |
| 2349916 | G | T |  | Intergenic 02555/02556 | Non-coding |
| 2349964 | G | – |  | Intergenic 02555/02556 | Non-coding |
| 2349972 | A | – |  | Intergenic 02555/02556 | Non-coding |
| 2349980 | T | – |  | Intergenic 02555/02556 | Non-coding |
| 2349986 | A | – |  | Intergenic 02555/02556 | Non-coding |
| 2349990 | A | – |  | Intergenic 02555/02556 | Non-coding |
| 2349995 | T | – |  | Intergenic 02555/02556 | Non-coding |
| 2350002 | T | – |  | Intergenic 02555/02556 | Non-coding |
| 2350004 | C | – |  | Intergenic 02555/02556 | Non-coding |
| 2350008 | A | – |  | Intergenic 02555/02556 | Non-coding |
| 2350012 | N | – |  | Intergenic 02555/02556 | Non-coding |
| 2350016 | A | – |  | Intergenic 02555/02556 | Non-coding |
| 2350100 | T | C |  | Intergenic 02555/02556 | Non-coding |
| 2383630 | G | T | Silent | 02591 | CHP, putative membrane protein |
| 2383660 | G | T | Silent | 02591 | CHP, putative membrane protein |
| 2420619 | – | T | Frame shift | 02632 | Teicoplanin resistance-associated membrane protein TcaB |
| 2446161 | C | G | Glu71Asn | 02662 | PTS system, sucrose-specific IIBC component ScrA |
| 2446162 | C | A | Gly70Val | 02662 | PTS system, sucrose-specific IIBC component ScrA |
| 2446164 | C | – | Frame shift | 02662 | PTS system, sucrose-specific IIBC component ScrA (02661 + 02662 = 1 orf) |
| 2446246 | C | T | Silent | 02662 | PTS system, sucrose-specific IIBC component ScrA |
| 2446393 | C | A |  | Intergenic 02662/02663 | Non-coding |
| 2446402 | C | – |  | Intergenic 02662/02663 | Non-coding |
| 2446423 | C | A |  | Intergenic 02662/02663 | Non-coding |
| 2446630 | C | T | His26Tyr | 02663 | CHP |
| 2446641 | T | A | Silent | 02663 | CHP |
| 2466536 | T | C | Lys349Glu | 02681 | Nitrate reductase, alpha subunit |
| 2556234 | A | G |  | Intergenic 02781/02782 | Non-coding |
| 2592013 | A | – | Frame shift | 02813 | Hypothetical membrane protein (02813 + 02814 = 1 orf) |
| 2596878 | C | T | Gly107Asp | 02818 | MFS family major facilitator transporter |
| 2678563 | T | C | Silent | 02911 | CHP |
| 2684051 | C | T | Ala90Thr | 02919 | 3-methyl-2-oxobutanoate hydroxymethyltransferase |
| 2689048 | G | T | Val353Leu | 02923 | Amino acid permease |
| 2782821 | C | – | Frame shift | 03008 | Imidazole glycerol phosphate synthase subunit HisF |

^1^Genome positions and locus numbers are according to NCTC8325 sequence (GenBank accession CP000253). ^2^Reference nucleotide in NCTC8325. ^3^Sequenced nucleotide in BB255, RA120 and ME51 determined in this study. ^4^Description of putative gene products and functions were taken from NCTC8325 annotations and were improved from annotations of other *S. aureus* strains. Abbreviation: CHP, conserved hypothetical protein.
